# Development and Validation of a Nomogram for Predicting Prognosis to Immune Checkpoint Inhibitors Plus Chemotherapy in Patients With Non-Small Cell Lung Cancer

**DOI:** 10.3389/fonc.2021.685047

**Published:** 2021-08-12

**Authors:** Hao Zeng, Wei-wei Huang, Yu-jie Liu, Qin Huang, Sheng-min Zhao, Ya-lun Li, Pan-wen Tian, Wei-min Li

**Affiliations:** ^1^Department of Respiratory and Critical Care Medicine, West China Hospital, West China School of Medicine, Sichuan University, Chengdu, China; ^2^Department of Respiratory and Critical Care Medicine, West China Hospital, Sichuan University, Chengdu, China; ^3^Department of Respiratory and Critical Care Medicine, Lung Cancer Treatment Center, West China Hospital, Sichuan University, Chengdu, China

**Keywords:** immune checkpoint inhibitors, chemotherapy, non-small cell lung cancer (NSCLC), nomogram, progression-free survival (PFS)

## Abstract

**Background:**

Immune checkpoint inhibitors (ICIs) plus chemotherapy improved the prognosis of patients with non-small cell lung cancer (NSCLC); however, reliable prognostic biomarkers are lacking. We explored factors associated with prognosis and developed a predictive model.

**Methods:**

We retrospectively analyzed 130 consecutive stage IIIA–IVB NSCLC patients treated with ICIs combined with chemotherapy. Cox univariate and multivariate proportional hazards regression analyses were used to identify prognostic factors associated with progression-free survival (PFS). A nomogram was developed based on key factors in the training cohort (*n* = 86) and evaluated in the validation cohort (*n* = 44). According to the nomogram-based total point scores, we divided patients into low- and high-risk groups.

**Results:**

In the training cohort, bone metastases (p = 0.017) and an increased derived neutrophil-to-lymphocyte ratio (p = 0.018) were significantly associated with poor PFS, while smoking (p = 0.007) and programmed death-ligand 1 (PD-L1) ≥50% (p = 0.001) were associated with improved PFS. A nomogram based on these factors was developed to predict PFS at 3, 6, and 12 months. The C-index of the nomogram to predict PFS was 0.725 (95% CI: 0.711–0.739) in the training cohort and 0.688 (95% CI: 0.665–0.711) in the validation cohort. The area under the curve (AUC) exhibited an acceptable discriminative ability, and calibration curves demonstrated a consistency between the actual results and predictions. In the training cohort, the median PFS (mPFS) was 12.3 and 5.7 months in the low- and high-risk groups, respectively (p < 0.001). In the validation cohort, the mPFS was 12.6 and 6.2 months in the low- and high-risk groups, respectively (p = 0.021).

**Conclusions:**

A predictive nomogram was developed to help clinicians assess prognosis early for advanced NSCLC patients who received ICI plus chemotherapy.

## Introduction

According to Global Cancer Statistics in 2020, lung cancer is the second most commonly diagnosed malignant tumor and remains the leading cause of cancer death in the world (1). Non-small cell lung cancer (NSCLC) accounts for approximately 85% of all lung cancers (2). In advanced NSCLC lacking actionable oncogenic drivers, platinum-based chemotherapy has traditionally been used as a treatment. However, the median progression-free survival (mPFS) time in response to this therapy is only 5–6 months (3). In recent years, considerable successes have been achieved using novel therapeutic strategies, i.e., immune checkpoint inhibitors (ICIs), as first-line and second-line treatments in patients with NSCLC (4, 5). Unfortunately, nearly 60% of patients with advanced NSCLC do not benefit from ICIs (6). Remarkable heterogeneity regarding their objective response rate, survival, immune-related adverse events (irAEs) in individual NSCLC patients, and limits in current biomarkers have driven some studies to look for new prognostic markers or to develop a comprehensive model to optimize patient benefit (7, 8).

Based on KEYNOTE-189, KEYNOTE-021, and KEYNOTE-407, the National Comprehensive Cancer Network (NCCN) guidelines recommend platinum-based chemotherapy plus ICIs as category 1 agents for first-line therapy in advanced NSCLC patients without actionable oncogenic drivers (4, 9–12). In clinical practice, the Chinese Experts Consensus made the same recommendation (13). However, in a subgroup analysis of PFS, a programmed death-ligand 1 tumor proportion score (PD-L1 TPS) <1% was not associated with PFS, which means that the level of PD-L1 expression was not entirely associated with the prognosis. PD-L1 as a predictive biomarker for patients treated with PD-1 inhibitors unfortunately remains complex, with inconsistent data between studies (4, 12, 14, 15). Similarly, there is no association between tissue tumor mutation burden (TMB) and efficacy for pembrolizumab plus chemotherapy or chemotherapy alone based on KEYNOTE-189, KEYNOTE-021, and KEYNOTE-407 (4, 10, 12). Some clinical characteristics and peripheral blood markers have been found to be related to the prognosis of patients treated with immunotherapy alone, such as liver or lung metastases, neutrophil-to-lymphocyte ratio (NLR), and derived NLR (dNLR) (6, 15–17). Based on NLR, serum albumin concentration, and lactate dehydrogenase (LDH), Lenci et al. developed a Gustave Roussy Immune (GRIm) score for advanced NSCLC patients treated with first-line pembrolizumab and showed that the low GRImT1 group had significantly longer PFS than the high GRImT1 group (18). However, the GRIm score only includes peripheral blood markers, and the utility for patients who receive chemoimmunotherapy is unknown. More comprehensive prognostic factors are needed. For example, in the real-world context, Cantini et al. even reported that high-intensity statins are associated with better PFS in advanced NSCLC patients treated with PD-1 inhibitors (19). There are currently limited biomarkers and no predictive model for patients with advanced NSCLC treated with PD-1 inhibitors plus chemotherapy. Therefore, it is necessary to explore biomarkers that are prognostic factors of these populations to identify patients who would benefit from chemoimmunotherapy.

We therefore conducted a study to assess prognostic factors in advanced NSCLC patients treated with PD-1 inhibitor plus chemotherapy. Finally, we aimed to develop a nomogram that is a reliable and convenient prognostic tool to quantify risk of progression for cancer patients (14, 16, 20) to accurately predict PFS.

## Material and Methods

### Patients

**Figure 1:**
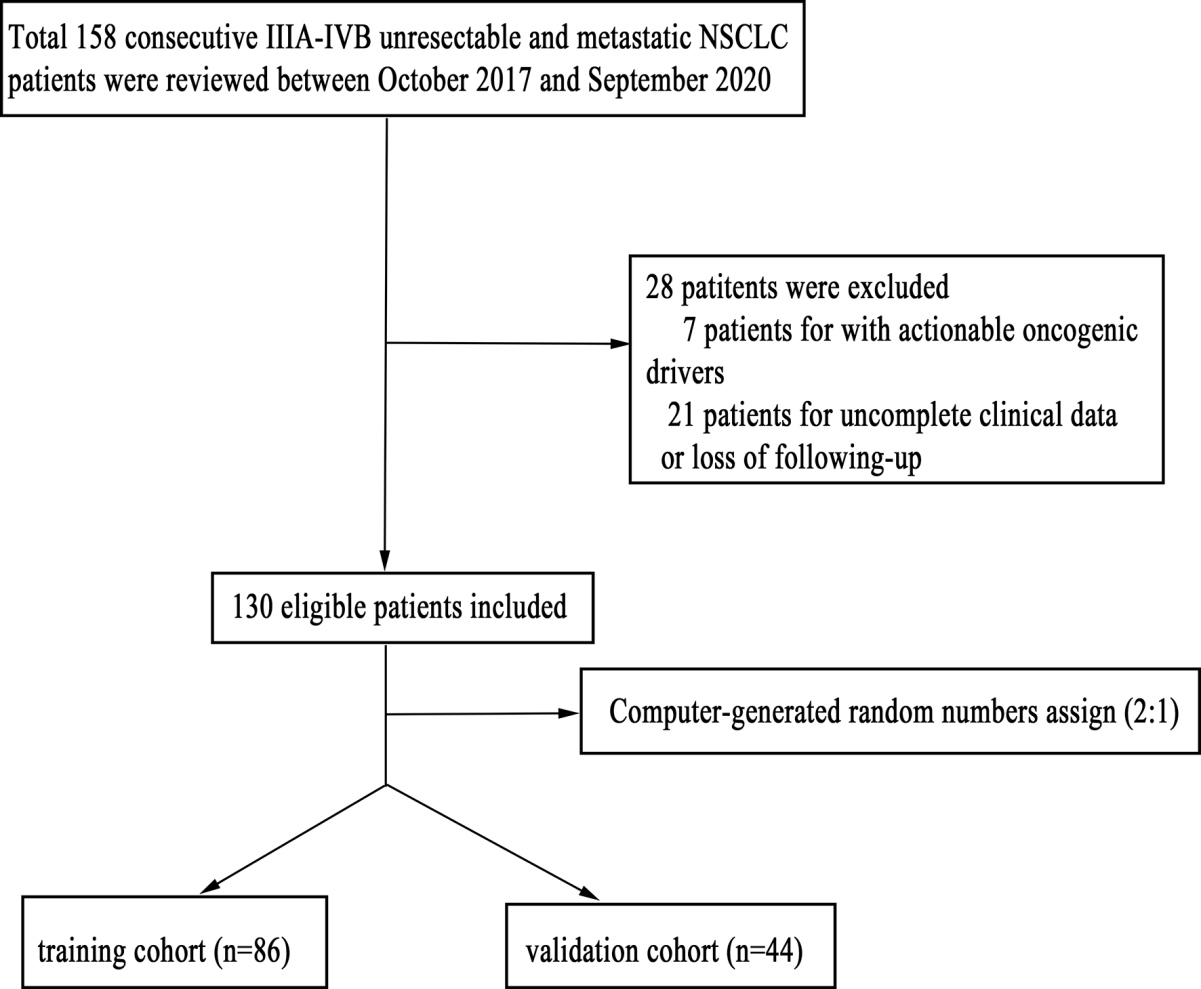
The workflow of patient selection.

We reviewed the electronic medical records of all patients with unresectable and metastatic (stage IIIA to IVB) NSCLC who received PD-1 inhibitor plus chemotherapy at West China Hospital between October 2017 and September 2020. A total of 158 consecutive patients were reviewed. The inclusion criteria were as follows: 1) pathologically confirmed NSCLC; 2) patients without actionable oncogenic drivers; and 3) patients with complete clinical data and follow-up information. Patients with other malignancies were excluded. Computer-generated random numbers were used to assign these patients into a training cohort and an internal validation cohort. The workflow of patient selection is shown in **Figure 1**. This study was approved by the Ethics Committee of West China Hospital (No. 2018-603), and the project was performed in accordance with the Declaration of Helsinki as revised in 2013.

### Data Collection

Data on clinical characteristics, laboratory parameters, and treatment information were extracted from the electronic inpatient record system of each patient and were updated as of February 1, 2021. Clinicopathological characteristics included sex, age, height, weight, clinical stage, smoking history, histology, metastatic sites, and PD-L1 expression level. Baseline (before the first injection of PD-1 inhibitor plus chemotherapy) peripheral blood indicators included LDH, red blood cell (RBC) count, hemoglobin (HB) count, platelet count, white blood cell (WBC) count, absolute neutrophil count (ANC), absolute lymphocyte count (ALC), absolute monocyte count (AMC), absolute eosinophil count (AEC), and carcinoembryonic antigen (CEA). Treatment records included the number of treatment lines, immunotherapy combined with chemotherapy regimens, and commencement/progression time of the treatment strategy. NLR = ANC/ALC, dNLR = ANC/(WBC − ANC) as defined previously (14).

### Treatment and Efficiency Assessment

Among the 130 patients, 88 patients were treated with pembrolizumab plus chemotherapy, 19 patients were treated with nivolumab plus chemotherapy, 13 patients were treated with sintilimab plus chemotherapy, five patients were treated with camrelizumab plus chemotherapy, two patients were treated with tislelizumab plus chemotherapy, two patients were treated with penpulimab plus chemotherapy, and one patient was treated with durvalumab plus chemotherapy (details shown in **Table S1**). The radiological response of tumors was evaluated using computed tomography every 8–10 weeks, and the radiologist was independent and blinded. Disease progression was evaluated according to the immune-related Response Evaluation Criteria in Solid Tumors Criteria (i-RECIST) (21). PFS was defined as the time from the date of treatment initiation until radiographic progression or death from any cause, whichever occurred first. Patients without radiographic disease progression on the date of the last follow-up were classified as censored.

### Statistical Analysis

The median and interquartile range (IQR) were used to describe continuous variables. Frequencies and percentages were used to describe categorical variables. Laboratory parameters were assessed as continuous variables. Age of 65 years was used as the cutoff point to convert age into a dichotomous variable. Body mass index (BMI) was divided into three groups according to Chinese standards (BMI < 18.5, underweight; 18.5 ≤ BMI < 24, normal; and BMI ≥ 24, overweight). Cox univariate and multivariate proportional hazards regression analyses were used to evaluate the impact of laboratory parameters and clinical characteristics on PFS. Variables with a p-value less than 0.1 in the univariate analysis were included in multivariate analysis. A two-tailed p-value of <0.05 was considered statistically significant.

In the training set, predictors derived from the multivariate Cox regression analysis were used to construct the nomogram and then validated in the validation cohort. Each nomogram was also validated internally by using bootstrap method with 1,000 resamples. The concordance index (C-index) and the area under the curve (AUC) were used to evaluate the discriminative ability of the nomogram. The first was computed in the Cox prediction models, while the second was obtained using receiver operating characteristic (ROC) curves with 3-, 6-, and 12-month PFS as binary outcomes. Calibration curves were used to compare the association between the actual outcomes and the predicted probabilities. Finally, we calculated the risk scores of all patients in the training set and validation set. We used X-tile software to select the cutoff point in the training set risk score, which was used to classify patients from the training set and validation set into two groups (low-risk group and high-risk group). The Cox proportional hazards regression model was used to compare whether the survival distributions differed between the two risk groups. Finally, we compared the current nomogram with Yuan’s nomogram (14), which was developed to predict prognosis in NSCLC patients treated with anti−PD−1 antibody, to demonstrate the advantage of the current nomogram to guide treatment decisions for patients treated with ICIs combined with chemotherapy. All statistical analyses were performed using SPSS version 24.0 Statistical Software (SPSS Inc., Chicago, IL, USA) and the R program (version 4.2.0).

## Results

### Patient Characteristics

We identified 158 consecutive IIIA–IVB unresectable and metastatic NSCLC patients who received PD-1 inhibitors plus chemotherapy. Of these, seven patients with actionable oncogenic drivers and 21 patients with incomplete clinical data or loss of follow-up were excluded, leaving 130 patients for analysis.

Among them, 86 patients were included in the model development cohort, and 44 were included in the validation cohort. Among 86 patients in the training cohort, the median age was 61.0 (53.0–68.0) years. Males accounted for 69 (80.23%), and smokers accounted for 67.44% of the subjects. Thirty (34.88%) patients had bone metastasis. Twenty (23.25%), 21 (24.42%), 23 (26.74%), and 22 (25.58%) patients had PD-L1 TPS <1%, ≥1%–49%, ≥50%, and unknown, respectively. The median dNLR was 2.19 (1.65, 3.30). Other clinical characteristics and laboratory parameters are shown in **Table 1**.

**Table 1:** Baseline clinical characteristics and laboratory parameters.

Variable	Training Set (*n* = 86)	Validation Set (*n* = 44)	Immunotherapy Combined With Chemotherapy Set (*n* = 130)
*Characteristics*			
Age, median, (25th, 75th)	61.0 (53.0, 68.0)	58.5 (51.2, 65.7)	61 (53.0, 67.0)
Gender, *n* (%)			
Male	69 (80.23)	33 (75.00)	102 (78.46)
Female	17 (19.77)	11 (25.00)	28 (21.54)
Smoking status, *n* (%)			
Never	28 (32.56)	17 (38.64)	45 (34.62)
Smoking	58 (67.44)	27 (61.36)	85 (65.38)
Diabetes or hypertension, *n* (%)			
No	59 (68.60)	32 (72.72)	91 (70.00)
Yes	27 (31.40)	12 (27.28)	39 (30.00)
BMI, *n* (%)			
<18.5	9 (10.47)	3 (6.81)	12 (9.23)
18.5-23.9	50 (58.14)	26 (59.09)	76 (58.46)
≥24	27 (31.39)	15 (34.10)	42 (32.31)
Histology, *n* (%)			
Squamous	34 (39.53)	21 (47.73)	55 (42.31)
Adenocarcinoma	50 (58.14)	19 (43.18)	69 (53.08)
Other NSCLC	2 (2.32)	4 (9.09)	6 (4.61)
Clinical stage, *n* (%)			
IIIA~IIIC	12 (13.95)	9 (20.45)	21 (16.15)
IV	74 (86.05)	35 (79.55)	109 (83.85)
N stage, *n* (%)			
N0~N1	19 (22.09)	6 (13.64)	25 (19.23)
N2~N3	67 (77.91)	38 (86.36)	105 (80.77)
Number of metastatic organs, *n* (%)			
≤1	49 (56.98)	27 (61.36)	76 (58.46)
>1	37 (43.02)	17 (38.64)	54 (41.54)
Metastatic, *n* (%)			
Brain	19 (22.09)	8 (18.18)	27 (20.77)
Liver	5 (5.81)	7 (15.91)	12 (9.23)
Bone	30 (34.88)	16 (36.36)	46 (35.38)
Adrenal	8 (9.30)	4 (9.09)	12 (9.23)
Pleural	20 (23.25)	15 (34.09)	35 (26.92)
Contralateral lung	33 (38.37)	13 (29.55)	46 (35.38)
Line of treatment, *n* (%)			
1	57 (66.28)	33 (75.00)	90 (69.23)
>1	29 (33.72)	11 (25.00)	40 (30.77)
Receipt of hormone treatment, *n* (%)			
No	26 (30.23)	18 (40.91)	44 (33.85)
Yes	60 (69.77)	26 (59.09)	86 (66.15)
PD-L1 TPS%, *n* (%)			
<1%	20 (23.25)	9 (20.46)	29 (22.30)
1%–49%	21 (24.42)	10 (22.73)	31 (23.85)
≥50%	23 (26.74)	8 (18.18)	31 (23.85)
Unknown	22 (25.58)	17 (38.64)	39 (30.00)
*Laboratory parameters (25th, 75th)*			
LDH (IU/L)	177 (150, 225)	172 (139, 226)	176 (146, 225)
RBC (×10^12^/L)	4.32 (4.03, 4.76)	4.23 (3.88, 4.66)	4.29 (3.93, 4.71)
HB (g/L)	130 (119, 142)	126 (112, 136)	128 (116, 141)
Platelet (×10^9^/L)	218 (166, 275)	245 (187, 314)	230 (171, 277)
WBC (×10^9^/L)	7.39 (5.45, 8.80)	7.06 (5.05, 9.15)	7.22 (5.38, 8.92)
ANC (×10^9^/L)	4.87 (3.60, 6.39)	5.12 (3.27, 6.80)	4.94 (3.50, 6.63)
AMC (×10^9^/L)	0.49 (0.37, 0.67)	0.51 (0.34, 0.66)	0.50 (0.36, 0.67)
ALC (×10^9^/L)	1.40 (0.91, 1.70)	1.50 (1.01, 2.00)	1.43 (0.96, 1.75)
AEC (×10^9^/L)	0.13 (0.05, 0.23)	0.14 (0.08, 0.29)	0.14 (0.07, 0.25)
dNLR	2.19 (1.65, 3.30)	2.40 (1.62, 3.56)	2.20 (1.65, 3.31)
NLR	3.54 (2.37, 5.83)	3.76 (2.39, 5.96)	3.54 (2.39, 5.83)
CEA (ng/ml)	5.27 (1.81, 24.75)	3.39 (1.87, 8.35)	3.81 (1.81, 13.97)

BMI, body mass index; NSCLC, non-non-small cell lung cancer; LDH, lactate dehydrogenase; RBC, red blood cell count; HB, hemoglobin; WBC, white blood cell count; ANC, absolute neutrophil count; AMC, absolute monocyte count; ALC, absolute lymphocyte count; AEC, absolute eosinophil count; dNLR, derived neutrophil-to-lymphocyte ratio; NLR, neutrophil-to-lymphocyte ratio; CEA, carcinoembryonic antigen.

After a median follow-up period of 11.1 months (range 6.5–18.4 months), at the last date of follow-up, the mPFS of the 130 patients was 9.2 (7.9, 10.4) months, and 21 patients died. The PFS probability in the whole patient population was 90.7%, 69.1%, and 30.3% at 3, 6, and 12 months, respectively.

### Independent Prognostic Factors in the Training Set

**Figure 2:**
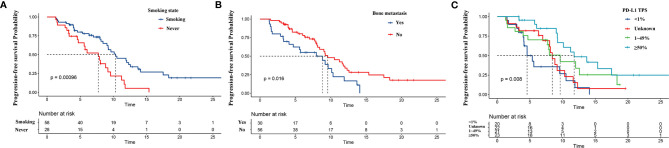
Kaplan–Meier curves for progression-free survival (PFS) based on predictors from the nomogram. PFS according to **(A)** smoking, **(B)** bone metastasis status, and **(C)** programmed cell death ligand-1 (PD-L1) expression.

The results of univariate and multivariate survival analyses of PFS are listed in **Table 2**. Univariate analysis showed that sex, smoking status, PD-L1 expression, bone metastasis, ALC, dNLR, and CEA were related to PFS (p < 0.1). Next, all significant factors in the univariate analysis were entered into the multivariate analysis, which indicated that bone metastasis (HR = 2.071, 95% CI: 1.138–3.766, p = 0.017) and higher dNLR (HR = 1.142, 95% CI: 1.023–1.275, p = 0.018) were significantly associated with shortened PFS, while smoking (HR = 0.419, 95% CI: 0.223–0.789, p = 0.007) and PD-L1 ≥50% (HR = 0.211, 95% CI: 0.087–0.509, p = 0.001) were independent protective factors for PFS. The Kaplan–Meier survival curve analysis showed that patients who developed bone metastasis and never smoked had a shortened PFS, and PD-L1 ≥50% was related to a prolonged PFS (**Figure 2**).

**Table 2:** Univariate and multivariate Cox analyses of progression-free survival (PFS).

Variables	Univariate Analysis		Multivariate Analysis	
	HR (95% CI)	p	HR (95% CI)	p
Gender				
Male	1 [Reference]			
Female	1.927 (1.037, 3.582)	0.038		
Age years				
≤65	1 [Reference]			
>65	0.797 (0.444, 1.433)	0.449		
BMI (kg/m^2^)		0.124		
<18.5	1 [Reference]			
18.5–23.9	0.659 (0.300, 1.446)	0.299		
≥24	0.417 (0.175, 0.996)	0.049		
Smoking status				
Never	1 [Reference]			
Smoking	0.397 (0.225, 0.700)	0.001	0.419 (0.223, 0.789)	0.007
Receipt of hormone treatment				
No	1 [Reference]			
Yes	0.946 (0.536, 1.670)	0.848		
Number of metastatic organs				
≤1	1 [Reference]			
>1	1.546 (0.904, 2.647)	0.112		
Clinical stage				
IIIA~IIIC	1 [Reference]			
IV	1.914 (0.817, 4.486)	0.135		
Histology		0.586		
Squamous	1 [Reference]			
Adenocarcinoma	1.345 (0.759, 2.386)	0.310		
Other NSCLC	1.398 (0.319, 6.123)	0.656		
Treatment lines				
1	1 [Reference]			
>1	0.856 (0.497, 1.475)	0.576		
PD-L1 TPS%		0.012		0.004
<1%	1 [Reference]			
Unknown	0.618 (0.296, 1.290)	0.200	0.543 (0.250, 1.178)	0.122
1%~49%	0.490 (0.229, 1.047)	0.065	0.473 (0.218, 1.023)	0.057
≥50%	0.271 (0.124, 0.593)	0.001	0.211 (0.087, 0.509)	0.001
Brain metastatic				
No	1 [Reference]			
Yes	1.578 (0.824, 3.022)	0.168		
Bone metastatic				
No	1 [Reference]			
Yes	1.955 (1.119, 3.417)	0.019	2.071 (1.138, 3.766)	0.017
Liver metastatic				
No	1 [Reference]			
Yes	1.624 (0.644, 4.094)	0.304		
Adrenal metastatic				
No	1 [Reference]			
Yes	0.822 (0.325, 2.077)	0.679		
Contralateral lung metastatic				
No	1 [Reference]			
Yes	0.707 (0.403, 1.240)	0.226		
Pleural metastatic				
No	1 [Reference]			
Yes	1.320 (0.704, 2.473)	0.387		
Diabetes or hypertension				
No	1 [Reference]			
Yes	0.680 (0.368, 1.258)	0.219		
N stage				
N0~N1	1 [Reference]			
N2~N3	0.956 (0.502, 1.820)	0.891		
LDH (IU/L)	1.001 (0.999, 1.003)	0.499		
RBC (×10^12^/L)	0.885 (0.591, 1.324)	0.553		
HB (g/L)	0.994 (0.981, 1.007)	0.394		
Platelet (×10^9^/L)	0.999 (0.996, 1.002)	0.640		
WBC (×10^9^/L)	1.022 (0.939, 1.113)	0.616		
ANC (×10^9^/L)	1.047 (0.964, 1.137)	0.281		
AMC (×10^9^/L)	0.335 (0.089, 1.258)	0.105		
ALC (×10^9^/L)	0.678 (0.426, 1.077)	0.090		
AEC (×10^9^/L)	0.822 (0.146, 4.622)	0.824		
dNLR	1.097 (0.999, 1.204)	0.053	1.142 (1.023, 1.275)	0.018
NLR	1.039 (0.983, 1.097)	0.176		
CEA (ng/ml)	1.002 (1.001, 1.004)	0.007		

BMI, body mass index; LDH, lactate dehydrogenase; RBC, red blood cell count; HB, hemoglobin; WBC, white blood cell count; ANC, absolute neutrophil count; AMC, absolute monocyte count; ALC, absolute lymphocyte count; AEC, absolute eosinophil count; dNLR, derived neutrophil-to-lymphocyte ratio; NLR, neutrophil-to-lymphocyte ratio; CEA, carcinoembryonic antigen.

### Establishment of a Prognostic Nomogram for Progression-Free Survival

**Figure 3:**
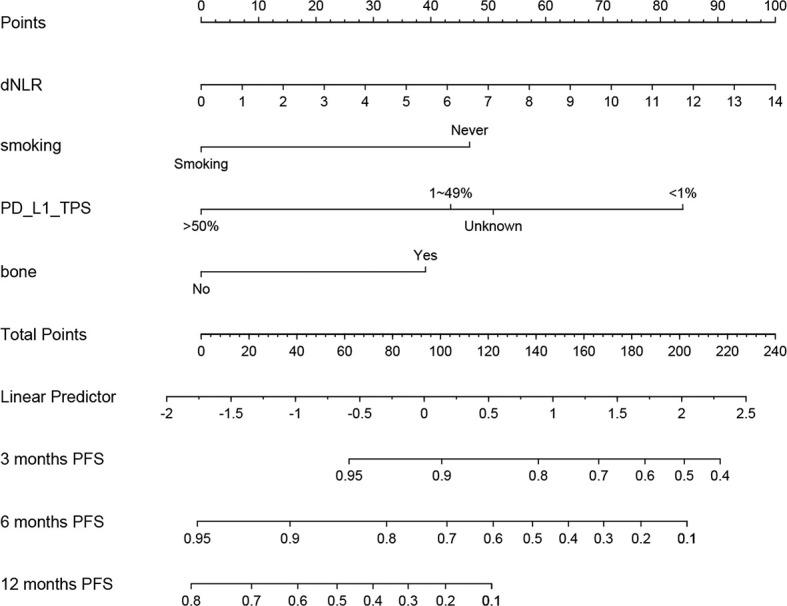
Prognostic nomogram for non-small cell lung cancer (NSCLC) patients treating immune checkpoint inhibitors (ICIs) plus chemotherapy to assign their probability of progression-free survival (PFS) at 3, 6, and 12 months.

According to predictive factors identified in the training cohort, we developed a nomogram to predict the probability of PFS at 3, 6, and 12 months in NSCLC patients treated with ICIs plus chemotherapy (**Figure 3**). Each prognostic parameter was assigned a corresponding number of risk points on the points scale. We obtained a total score by delineating a vertical line and summing the corresponding risk points of each parameter. Finally, we drew a vertical line towards the PFS probability axis, which could help to estimate the specific probability of PFS at 3, 6, and 12 months for each specific NSCLC patient.

### Evaluation and Validation of the Nomogram

**Figure 4:**
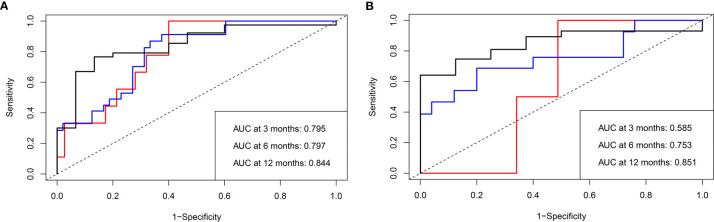
Receiver operating characteristic (ROC) curves of the nomogram to predict progression-free survival (PFS) in both the training and validation cohorts. The area under the curve (AUC) of the probability of PFS at 3, 6, and 12 months in **(A)** the training and **(B)** validation cohorts, respectively.

**Figure 5:**
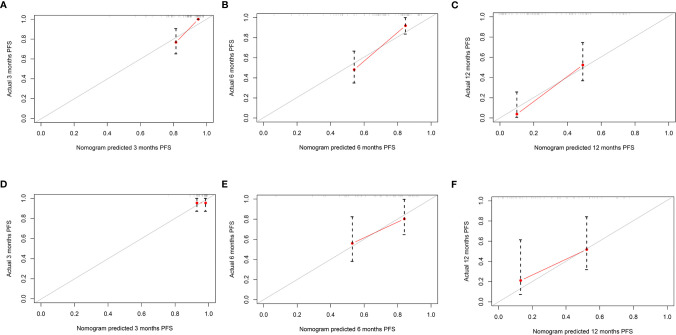
The calibration curves of the nomogram for predicting progression-free survival (PFS) in both the training and validation cohorts. The x-axis represents the nomogram predicted probability, and the y-axis represents the actual probability of PFS. The red line indicates the performance of the nomogram, of which a closer fit to the gray line represents a better prediction. Calibration curves of the nomogram for predicting PFS at **(A)** 3, **(B)** 6, and **(C)** 12 months in the training cohort. Calibration curves of the nomogram to predict PFS at **(D)** 3, **(E)** 6, and **(F)** 12 months in the validation cohort.

The mPFS was 9.6 months (95% CI: 7.1, 12.1 months) in the validation set. The C-index of the nomogram to predict PFS was 0.725 (95% CI: 0.711–0.739) in the training cohort and 0.688 (95% CI: 0.665–0.711) in the validation cohort. In addition, the AUCs of the nomogram to predict PFS at 3, 6, and 12 months were 0.80 (95% CI: 0.66–0.91), 0.80 (95% CI: 0.69–0.89), and 0.84 (95% CI: 0.74–0.96) in the training cohort and 0.59 (95% CI: 0.41–0.75), 0.75 (95% CI: 0.57–0.93), and 0.85 (95% CI: 0.70–1.00) in the validation cohort, respectively (**Figure 4**). Additionally, there was good consistency between the actual outcomes and the predicted outcomes according to the calibration curves in the training cohort and validation cohort (**Figure 5**).

**Figure 6:**
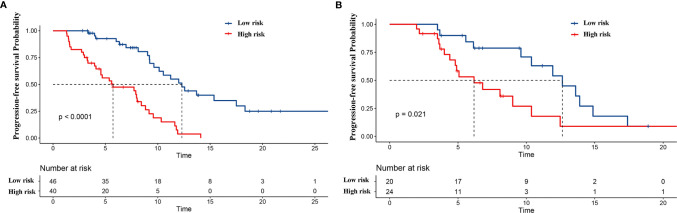
Kaplan–Meier curves for two subgroups according to the cutoff value in the training cohort of the nomogram-based total score. The blue curve and red curve represent the low-risk (0–100) and high-risk (>100) groups, respectively. Kaplan–Meier curves for progression-free survival (PFS) in **(A)** the training cohort and **(B)** validation cohort.

Furthermore, patients in the training set and validation set were divided into two subgroups according to the cutoff value of the nomogram-based total score: the low-risk group (0–100) and the high-risk group (>100). In the training set, 46 patients were assigned to the low-risk group, while 40 patients were assigned to the high-risk group. The Kaplan–Meier survival curve analysis showed that the mPFS was 12.3 (95% CI: 9.8, 14.9) months and 5.7 (95% CI: 1.7, 9.8) months in the low-risk group and high-risk group (p < 0.001), respectively. In the validation set, 20 patients were assigned to the low-risk group, while 24 patients were assigned to the high-risk group. The mPFS was 12.6 (95% CI: 9.2, 16.1) months and 6.2 (95% CI: 3.7, 8.7) months in the low-risk and high-risk groups (p = 0.021), respectively (**Figure 6**). Cox univariate regression analysis showed that patients in the high-risk group had a shortened PFS (HR = 4.726, 95% CI: 2.579–8.659, p < 0.001). Similarly, the high-risk group was linked to a shortened PFS in the validation set (HR = 2.422, 95% CI: 1.113–5.270, p = 0.026).

### Comparison of Current Nomogram and Previous Nomogram

**Figure 7:**
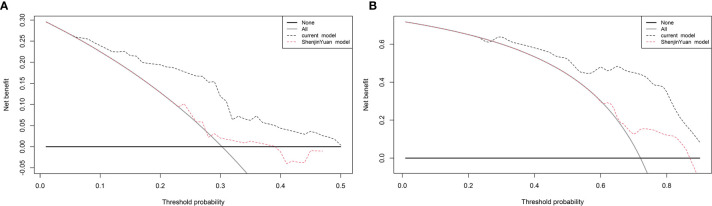
Decision curve analysis for progression-free survival (PFS). The straight gray line represents the assumption that all patients will progress at **(A)** 6 and **(B)** 12 months, and the black horizontal line represents the assumption that no patients will progress at **(A)** 6 and **(B)** 12 months.

We compared the current nomogram model with Yuan’s nomogram, which was developed to predict NSCLC patients treated with anti−PD−1 antibody. Decision curve analysis for 6- and 12-month PFS revealed that the current nomogram had a higher benefit (**Figure 7**).

## Discussion

Immunotherapy is widely used in the treatment of NSCLC patients who lack targetable aberrations and show improved efficacy over standard platinum-based doublet chemotherapy (22). Immunotherapy combined with chemotherapy has been recommended as category 1 for treating advanced NSCLC patients in the NCCN guidelines (9). Some studies have shown that pembrolizumab combined with chemotherapy has a better PFS than pembrolizumab alone for treating NSCLC patients (23, 24). However, studies about prognostic factors associated with shorter PFS for immunotherapy plus chemotherapy individuals are limited. This is the first study to develop a comprehensive model that incorporates PD-L1, easily accessible clinical characteristics, serum parameters, and imaging features to predict the probability of PFS at 3, 6, and 12 months for NSCLC patients treated with ICIs plus chemotherapy. Here, we identified four factors, including dNLR, smoking history, PD-L1 TPS, and bone metastasis, which were associated with PFS in this population.

Some recent papers about NLR, inflammation-related peripheral blood parameters, have been reported and have shown that increased NLR is associated with worsened prognosis in patients receiving immunotherapy (25). The lung immuno-oncology prognostic score (LIPS-3), which includes NLR, PD-L1 tumor cell expression level, and LDH, was developed to classify NSCLC patients with PD-L1 ≥50% who received first-line pembrolizumab and showed that NLR <4 was a significant prognostic factor (26). Similarly, some studies showed that a high dNLR was associated with poorer prognosis in patients treated with immunotherapy alone (27–29). Inflammation plays an important role in tumor development, affecting the survival of cancer patients. dNLR, a novel index derived from NLR, may reflect cancer-associated inflammation and determine disease progression (27). Mezquita et al. (27) showed that a baseline dNLR >3 was independently associated with PFS in patients with advanced NSCLC treated with PD-1/PD-L1 inhibitors. Additionally, Yuan et al. (14) showed that a high dNLR was associated with a poorer OS and developed a nomogram that incorporated dNLR to predict prognosis of NSCLC patients treated with anti−PD−1 antibodies. In our present study, we found that increased dNLR was also correlated with poor outcomes with ICI plus chemotherapy.

Regarding clinical characteristics, our results showed that smoking was an independent protective factor for NSCLC patients treated with ICIs plus chemotherapy. Similarly, a previous study showed that current/former smokers experienced improved PFS and OS when PD-L1 expression ≥50% and first-line pembrolizumab was administered (30). Additionally, two studies aiming to develop nomograms to predict the prognosis of NSCLC patients treated with anti-PD-1 inhibitors both showed that smoking was associated with improved prognosis and incorporated it into the model (8, 14). Smokers were more likely to exhibit positive PD-L1 expression and higher TMB, which may improve the therapeutic efficacy of PD-1 inhibitors (31). The potential mechanism involved recruitment of tumor-infiltrating lymphocytes (TILs) and release of interferon-γ (IFN-γ) under a chronic inflammatory microenvironment caused by tobacco exposure, which induced PD-L1 expression and enhanced the stability of PD-L1 (8). Limited data from a meta-analysis indicated that both smokers and nonsmokers benefit from chemoimmunotherapy (32, 33), but our retrospective study yielded different PFS rates between smokers and never smokers.

To date, although its expression may vary over time and by site, PD-L1 expression is the only approved predictive biomarker for PD-(L)1 blockade in NSCLC. Not only the NCCN guidelines but also the American Society of Clinical Oncology (ASCO) and Ontario Health Cancer Care (CCO) NSCLC expert panels made recommendations for therapy for patients without driver alterations based on PD-L1 expression (9, 34). Our study demonstrates that high PD-L1 expression is related to prolonged PFS in NSCLC patients treated with ICIs plus chemotherapy.

A few studies have investigated the prognostic role of metastatic sites of disease in NSCLC patients treated with ICIs. Pantano et al. showed that the number of liver metastases is significantly correlated with time-to-treatment failure, while there was no statistically significant difference for bone metastases (35). The incidence of bone metastases in NSCLC is 20%–40% (32). Bone marrow, a well-known secondary lymphatic organ, hosts several immune cells that are potentially able to affect systemic immunity and the therapeutic efficacy of immunotherapy (36, 37). In a retrospective study of NSCLC patients receiving nivolumab, patients with bone metastasis had significantly reduced PFS than patients without, which was similar to a study of patients receiving pembrolizumab showing that patients with bone metastasis exhibited significantly shorter PFS (37, 38). However, in another retrospective study of advanced NSCLC treated with pembrolizumab, bone metastasis did not affect PFS, which may be related to the relatively small sample size (39). In a real-life study, bone metastases were a general prognostic factor in NSCLC patients, regardless of the treatment; and most studies indicate that patients with bone metastases experience significantly shorter PFS (37, 40). For patients treated with ICIs plus chemotherapy, our study also demonstrated that bone metastasis was linked to a shortened PFS.

The C-index, AUC, and calibration curves implied the predictive accuracy of the current model as reported models, but the AUC of the nomogram to predict PFS at 3 months was relatively low in the validation cohort, which means that the model had a weak ability to predict PFS at 3 months but was better able to predict PFS at 6 and 12 months (14, 41). We attribute this to several causes. First, there was a relatively small sample size in the validation cohort. Second, as many studies have reported, the median time of irAEs is approximately 3 months, which may lead to changes in treatment regimens or even interruptions (42–44). The current nomogram model was compared with Yuan’s model, which was developed to predict the prognosis of NSCLC patients treated with anti−PD−1 antibody (14). The current nomogram revealed an advantage in predicting the PFS of NSCLC patients treated with ICIs plus chemotherapy. To the best of our knowledge, this is the first nomogram based on PD-L1, clinical characteristics, laboratory parameters, and imaging features for predicting the prognosis of patients treated with ICI plus chemotherapy.

There are several limitations to our study. First, this was a retrospective study of a single center with a small population and lacks external validation. Larger-scale and multicenter prospective studies are needed to validate our findings. Second, this study only focused on PFS in NSCLC patients treated with PD-1 inhibitors plus chemotherapy due to the short follow-up time. Finally, this study lacks some other important predictive biomarkers, such as TILs, TMB, human leukocyte antigen (HLA), and broadly predictors, which should be explored in the future.

In conclusion, the novel nomogram model based on comprehensive factors has acceptable predictive accuracy and discriminative ability and could be applied to estimate the probability of PFS in advanced NSCLC patients treated with ICIs plus chemotherapy, especially for 6 and 12 months, and will assist clinicians in guiding treatment decisions in clinical practice. But larger samples, multicenter prospective studies, and external validation are still needed to develop a better nomogram for these populations.

## Data Availability Statement

The original contributions presented in the study are included in the article/**Supplementary Material**. Further inquiries can be directed to the corresponding authors.

## Ethics Statement

The study was approved by the Ethics Committee of West China Hospital (No. 2018-603). Written informed consent for participation was not required for this study in accordance with the national legislation and the institutional requirements.

## Author Contributions

HZ, W-wH, Y-jL, Y-lL, and P-wT contributed to the conception and design of the study. HZ, QH, S-mZ, Y-lL, and PT organized the database. HZ, W-wH, QH, and S-mZ performed the statistical analysis. HZ and W-wH wrote the first draft of the manuscript. HZ and W-wH contributed to this study equally. All authors contributed to the article and approved the submitted version.

## Funding

This work was supported by the National Science Foundation of China (82072598, 81871890, and 91859203), the National Major Science and Technology Project of China (2017ZX10103004-012), the National Key Development Plan for Precision Medicine Research of China (2017YFC0910004), the Science and Technology Program of Sichuan, China (2020YFS0572), and the Major Science and Technology Innovation Project of Chengdu City, China (2020-YF08-00080-GX).

## Conflict of Interest

The authors declare that the research was conducted in the absence of any commercial or financial relationships that could be construed as a potential conflict of interest.

## Publisher’s Note

All claims expressed in this article are solely those of the authors and do not necessarily represent those of their affiliated organizations, or those of the publisher, the editors and the reviewers. Any product that may be evaluated in this article, or claim that may be made by its manufacturer, is not guaranteed or endorsed by the publisher.
