# Supplementary material for: Development and Validation of a Nomogram for Predicting Prognosis to Immune Checkpoint Inhibitors Plus Chemotherapy in Patients With Non-Small Cell Lung Cancer
Source: Front Oncol. 2021 Aug 12;11:685047. doi: 10.3389/fonc.2021.685047 (PMC8397581; doi:10.3389/fonc.2021.685047)
Supplement: Supplementary file 1 [file Table_1.docx]

| **Table S1 Number of people receiving chemotherapy plus immunotherapy** | | |
| --- | --- | --- |
| Treatment | Training set | Validation set |
| Pembrolizumab+AC | 22 | 8 |
| Pembrolizumab+A | 4 | 2 |
| Pembrolizumab+AP | 5 | 3 |
| Pembrolizumab+GP | 0 | 1 |
| Pembrolizumab+T | 6 | 1 |
| Pembrolizumab+TC | 14 | 12 |
| Pembrolizumab+TP | 5 | 3 |
| Pembrolizumab+Docetaxel | 2 | 0 |
| Nivolumab+Docetaxel | 1 | 3 |
| Nivolumab+DN | 1 | 0 |
| Nivolumab+GN | 1 | 0 |
| Nivolumab+AP | 0 | 1 |
| Nivolumab+A | 0 | 1 |
| Nivolumab+P | 0 | 1 |
| Nivolumab+AC | 3 | 0 |
| Nivolumab+TC | 4 | 0 |
| Nivolumab+T | 1 | 0 |
| Nivolumab+TP | 1 | 0 |
| Nivolumab+GP | 1 | 0 |
| Camrelizumab+TC | 0 | 1 |
| Camrelizumab+AC | 4 | 0 |
| Sintilimab+TC | 1 | 4 |
| Sintilimab+AP | 0 | 1 |
| Sintilimab+AC | 6 | 0 |
| Sintilimab+A | 1 | 0 |
| Tislelizumab+TC | 1 | 1 |
| Penpulimab+TC | 1 | 1 |
| Durvalumab+GC | 1 | 0 |

Abbreviation: A, Pemetrexed; C, Carboplatin; T, Paclitaxel; P, Cisplatin; D, Docetaxel; N, Nedaplatin; G, Gemcitabine.
